# 

**Published:** 1904-02-27

**Authors:** 


### Publications.

Dr. Otto Schmidt's

**SPECIFIC TREATMENT OF CANCER.**

A Critique Based on Personal Observation.

By JOHN SHAW, M.D.Lond., M.R.C.P.Lond.

Price 1/- net.

London: H. J. GLAISHER, 57 Wigmore St., Cavendish Sq., W.

---

Second Edition. Crown 8vo., price 3/6 net.

With original "Plea—1900—for the State Registration of Nurses."

**MOCK NURSES OF THE LATEST FASHION.**

By FREDERICK J. GANT, F.R.C.S.,
Consulting Surgeon to the Royal Free Hospital.

BAILLIÈRE, TINDALL & COX,
Henrietta Street, Covent Garden, London.

---

Crown 8vo. Illustrated. Price **2s. 6d.** net.

**ANÆSTHETICS.** By J. BLUMFELD,

M.D.Cantab., Senior Anæsthetist to St. George's Hospital; Anæsthetist to the Grosvenor Hospital for Women and Children, London, and to the National Dental and Metropolitan Hospitals.

*Lancet.*—"....In writing this handbook the author has been quite successful." *Australasian Medical Gazette.*—"We can strongly commend this book." *Medical Chronicle.*—"The book is one which we can heartily recommend to senior students and practitioners."

London: BAILLIÈRE, TINDALL & COX, 8 Henrietta St., Covent Garden.

### NOTICE TO CORRESPONDENTS.

All MSS., Letters, Books for Review, and other matters intended for the Editor should be addressed The Editor, "The Hospital," The Hospital Buildings, 28 & 29 Southampton Street, Strand, W.C.

The Editor cannot undertake to return rejected MS., even when accompanied by stamped directed envelope.

**Notice to Advertisers and Subscribers.**

All Advertisements, Orders for Copies of The Hospital, and Business Communications should be addressed to **THE MANAGER (Not to the Editor),** The Hospital, 28 & 29 Southampton Street, Strand, London, W.C.

The Cost of Subscription, post free, is as follows:—Per week, 3½d.; per quarter, 3s. 9d.; per half-year, 7s. 6d.; per annum, 15s. Foreign and Colonial:—Per annum, 19s. 6d., payable, in all cases, in advance.

**NOTICE.—Vol. XXXIV. of "The Hospital" (April 1903 to September 1903), in handsome cloth binding, is now on sale at the office of this Journal, price 10s. 6d. Cases for binding the Numbers contained in this Volume 2s. Index to Vol. XXXIV., 2½d., post free.**

**The Monthly part for March will be ready on March 1st. Price 1s., by post, 1s. 3d.**

AN illustrated pamphlet has been issued in commemoration of the centenary of the Royal London Ophthalmic Hospital, in which is given a brief history of that institution. The hospital was founded in 1804, one of the chief circumstances which brought this about being the great prevalence of ophthalmia at that time in London, due, it is said, to the return of soldiers from Egypt where they had suffered severely from that complaint. The present building, which provides 138 beds for in-patients and a large out-patient department, was opened in 1899 by the Prince and Princess of Wales. The number of patients relieved annually has grown to such an extent that an additional £6,000 in annual subscriptions and donations is now required to meet the necessary expenditure.

### THE STUDENT OF MEDICINE.

### APPOINTMENTS.

**H. T. D. Acland**, F.R.C.S.Eng., Honorary Surgeon to the Christchurch Hospital, New Zealand. **J. A. Bowes**, M.D., B.C.Cantab., Certifying Factory Surgeon for the Herne Bay District, county Kent. **O. Burkitt**, L.R.C.P.&S.I., Ear and Throat Surgeon, Perth Hospital, West Australia. **R. B. Campbell**, M.B., C.M.Edin., Medical Superintendent of the Inverness District Asylum. **T. G. Davy**, M.R.C.S., Physician to In-patients, Perth Hospital, West Australia. **Lachlan Grant**, M.D., C.M.Edin., Certifying Factory Surgeon for the Ballachulish District, county Argyll. **Matthew Harris**, M.B.Aberd., Physician to the Wee Waa Hospital, New South Wales. **A. S. Hedley**, M.B., B.S.Durh., Certifying Factory Surgeon for the Rothbury District, county Northumberland. **Frances Ivens**, M.S.Lond., Surgical Assistant to the New Hospital for Women. **H. A. Leschen**, M.B., Ch.M.Edin., Physician to In-patients, Perth Hospital, West Australia. **F. J. Lovegrove**, M.R.C.S.Eng., L.S.A., Government Medical Officer and Vaccinator at Gunning, New South Wales. **J. Cole Marshall**, M.B., F.R.C., Clinical Assistant to the Chelsea Hospital for Women. **S. H. R. Montgomery**, M.B., B.S.R.U.I., Inspector-General of the Insane, Western Australia. **Edward H. Morgan**, M.R.C.S., Health Officer for the Hamilton District, Tasmania. **G. Watson**, M.B., Ch.M.Edin., Health Officer for the Burnie District, Tasmania.

**"The Hospital" Institutional Library.**

**"Hospitals and Asylums of the World." 4 Vols., with a Portfolio of Plans, complete £12 12s.**

**"Burdett's Hospitals and Charities, 1903." 5s. net.**

**"Burdett's Official Nursing Directory, 1903." 3s. net.**

**"Cottage Hospitals, General, Fever, and Convalescent." 10s. 6d.**

**"Uniform System of Accounts for Hospitals and Public Institutions." New Edition. 4s. net.**

**"Hospital Expenditure: The Commissariat." 2s. 6d.**

**"A Legal Handbook for the Use of Hospital Authorities." 2s. 6d.**

**"The Cottage Hospital Case Book or Register of Patients." 10s. and 12s. 6d.**

**All these are published by the SCIENTIFIC PRESS, Ltd., and may be obtained through any bookseller, or direct from the publishers, 28 and 29 Southampton Street, Strand, London, W.C.**

Crown 8vo. Illustrated. Price 1/- net.

THE

**RADICAL CURE OF CORNS AND BUNIONS.**

By E. HARDING FREELAND, F.R.C.S.,

Surgeon to the St. George's and St. James's Dispensary, &c.

"Mr. Freeland has done good service by affording, through proper and professional means, good prospects of relief from the troublesome and often disabling effects of these minor ailments."—*Brit. Med. Jour.*

London: BALE, SONS & DANIELSSON, 83 Gt. Titchfield Street, W.

**MEDICAL HISTORY FROM THE EARLIEST TIMES.**

By E. T. WITHINGTON, M.A., M.B. Oxon.
**Demy 8vo., over 400 pp., with two Plates, cloth gilt, 12/6 net.**

"One of the best attempts that has yet been made in the English language to present the reader with a concise epitome of the history of medicine, and as such we very cordially commend it." *Glasgow Medical Journal.*

THE SCIENTIFIC PRESS, LTD.,
28 and 29 Southampton Street, Strand, London, W.C.

### STANDARD NURSING MANUALS.

| | | |
|---|---|---|
| 5/- net, 5/4 Post Free. | **A HANDBOOK FOR NURSES.** By J. K. Watson, M.D., M.B., C.M. New and Revised Edition. Crown 8vo. profusely illustrated, cloth gilt - - - - - - - | 5/- net, 5/4 Post Free. |
| 3/6 net, 3/10 Post Free. | **SURGICAL WARD-WORK AND NURSING.** By Alexander Miles, M.D.Edin., C.M., F.R.C.S.E. Second Edition, Enlarged and entirely Revised. Demy 8vo. copiously illustrated, cloth gilt - - - - | 3/6 net, 3/10 Post Free. |
| 3/6 net, 3/10 Post Free. | **OPHTHALMIC NURSING.** By Sydney Stephenson, M.B., F.R.C.S.E. New and Revised Edition. Crown 8vo. profusely illustrated with Original Drawings, list of Instruments required, and a Glossary of Terms, cloth - - - | 3/6 net, 3/10 Post Free. |
| 2/- Post Free. | **PRACTICAL GUIDE TO SURGICAL BANDAGING AND DRESSINGS.** By Wm. Johnson Smith, F.R.C.S. Demy 16mo. profusely illustrated (suitable for Apron pocket), cloth - - - - - - - - - | 2/- Post Free. |
| 2/6 Post Free. | **HOSPITAL SISTERS AND THEIR DUTIES.** By Eva C. E. Lückes, Matron, London Hospital. Third Edition, Enlarged and partly re-written. Crown 8vo. about 200 pp. cloth gilt - - - - - | 2/6 Post Free. |
| 1/- net, 1/1 Post Free. | **THE EXAMINATION OF THE URINE.** By J. K. Watson, M.D., M.B., C.M., Author of "A Handbook for Nurses." Demy 16mo. cloth boards - - - - - - | 1/- net, 1/1 Post Free. |
| 2/6 Post Free. | **NURSING IN DISEASES OF THE THROAT, NOSE AND EAR.** By P. Macleod Yearsley, F.R.C.S.Eng., M.R.C.S., L.R.C.P.Lond. Demy 8vo. illustrated, cloth - - - - - - | 2/6 Post Free. |

*Of all Booksellers, or of*

THE SCIENTIFIC PRESS, LTD., 28 & 29 Southampton Street, Strand, London, W.C.

*Complete Catalogue of Nursing Handbooks post free on application.*

